# Elements of Physiology

**Published:** 1842-12-24

**Authors:** 


*Elements of Physiology.* By J. Müller, M. D., Professor of Anatomy in the University of Berlin, etc. *Translated from the German.* By Wm. Baly, M. D., Graduate in Medicine in the University of Berlin. *Arranged from the Second London Edition.* By John Bell, M. D., etc., etc. Philadelphia: 1843. 8 vo. pp. 886.

The established reputation of the great work of Müller requires no praise from us. It will long endure as an evidence of immense research, careful observation, patient investigation, untiring industry, and unrivalled ability. We hailed its incorporation into our own medical literature by the translation of Dr. Baly, and believed that in so doing he conferred an inestimable benefit on the profession generally. We long anticipated its republication in this country as a tribute to the great genius of its author. We did not, we confess, ever indulge in the utopian revery that it would become popularized—that it would be the vade mecum of the general practitioner; but we did hope to see it in most libraries, as a work of frequent reference. We, therefore, cannot but regret, and deeply, too, the mutilation which it has undergone, unjustifiable as it is, both morally and as a matter of expedient. No one will understand it more perfectly in its present emasculated state, and this continued appropriation of the property of others, this Procrustian system of adaptation to the narrow, sordid views of publisher or editor, is more than of doubtful morality. The scissors and the paste brush have taken the place of the mind and the pen, and the untiring exertions of the steam press can hardly keep pace with the voracious demands of the book-wright.

